# Global prevalence and epidemiological trends of Hashimoto's thyroiditis in adults: A systematic review and meta-analysis

**DOI:** 10.3389/fpubh.2022.1020709

**Published:** 2022-10-13

**Authors:** Xiaojie Hu, Yuquan Chen, Yiting Shen, Rui Tian, Yuqin Sheng, Huafa Que

**Affiliations:** ^1^Department of Traditional Chinese Surgery, Longhua Hospital Affiliated to Shanghai University of Traditional Chinese Medicine, Shanghai, China; ^2^Longhua Medical College, Shanghai University of Traditional Chinese Medicine, Shanghai, China; ^3^Institute of Medical Information/Medical Library, Chinese Academy of Medical Sciences & Peking Union Medical College, Beijing, China

**Keywords:** Hashimoto's thyroiditis, global prevalence, epidemiological trends, systematic review, meta-analysis

## Abstract

**Objective:**

Although Hashimoto's thyroiditis is associated with cardiovascular disease and malignancy, the global status of Hashimoto's thyroiditis is not well characterized across regions. Our objective was to evaluate the prevalence and trends of Hashimoto's thyroiditis in adults in regions with different economic income levels around the world.

**Methods:**

For this systematic review and meta-analysis, we searched PubMed, Embase, MEDLINE, Scopus, and Web of Science databases, and 48 random-effects representative studies from the inception to June 2022 were included without language restrictions to obtain the overall prevalence of Hashimoto's thyroiditis in adults worldwide. In addition, we stratified by time of publication, geographic region, economic level of the region of residence, gender, diagnostic method, etc.

**Results:**

A total of 11,399 studies were retrieved, of which 48 met the research criteria: 20 from Europe, 16 from Asia, five from South America, three from North America, and three from Africa. Furthermore, there are two projects involving 19 countries and 22,680,155 participants. The prevalence of Hashimoto's thyroiditis was 7.5 (95%CI 5.7–9.6%), while in the low-middle-income group the prevalence was 11.4 (95%CI 2.5–25.2%). Similarly, the prevalence was 5.6 (95%Cl 3.9–7.4%) in the upper-middle-income group, and in the high-income group, the prevalence was 8.4 (95%Cl 5.6–11.8). The prevalence of Hashimoto's varied by geographic region: Africa (14.2 [95% CI 2.5–32.9%]), Oceania (11.0% [95% CI 7.8–14.7%]), South America and Europe 8.0, 7.8% (95% Cl 0.0–29.5%) in North America, and 5.8 (95% Cl 2.8–9.9%) in Asia. Although our investigator heterogeneity was high (I^2^), our results using a sensitivity analysis showed robustness and reliability of the findings. People living in low-middle-income areas are more likely to develop Hashimoto's thyroiditis, while the group in high-income areas are more likely to develop Hashimoto's thyroiditis than people in upper-middle-income areas, and women's risk is about four times higher than men's.

**Conclusions:**

Global Hashimoto's thyroiditis patients are about four times as many as males, and there are discrepancies in the regions with different economic levels. In low-middle-income areas with a higher prevalence of Hashimoto's thyroiditis, especially countries in Africa, therefore local health departments should take strategic measures to prevent, detect, and treat Hashimoto's thyroiditis. At the same time, the hidden medical burden other diseases caused by Hashimoto's thyroiditis should also be done well.

**Systematic review registration:**

https://www.crd.york.ac.uk/prospero/, identifier: CRD 42022339839.

## Introduction

Hashimoto's thyroiditis (HT), also known as autoimmune thyroiditis (AIT) or chronic lymphocytic thyroiditis, is an autoimmune disease of the thyroid gland, often characterized by an enlarged thyroid gland, lymphocytic infiltration, and elevated serum autoimmune antibody levels. HT is a common cause of hypothyroidism in iodine-replete settings and increases the risk of malignancy (1–3).

The prevalence of HT varies by region and socioeconomic level, ranging from 4.8–25.8% in women and 0.9–7.9% in men (4). As we all known, the prevalence of HT varies significantly depending on geographic location. Although several previous studies have systematically described the prevalence of HT, none of them reviewed global HT prevalence and trends. Current researches have shown that the global prevalence of various autoimmune diseases was increasing (5), it is worth being exploring whether the global prevalence of HT has also increased. This study aimed to quantify the possible healthcare burden and plan for the future by assessing the global prevalence and trends of HT by analyzing the prevalence of HT in different regions.

## Methods

### Registration

Meta-based analysis was applied in this study. Compared with traditional literature review or the emerging bibliometric analysis, systematic review and meta-analysis had a relatively broad horizon of the current hotspots and can quantitatively reflect the research status in the field (1–3). This systematic review was conducted in accordance with the Preferred Reporting Items for Systematic Reviews and Meta-Analysis Protocols guidelines (4) and is registered with the International Prospective Register of Systematic Reviews (CRD42022339839).

### Search strategy and selection criteria

Five database including PubMed, Embase, MEDLINE, Scopus, and Web of Science databases were searched from inception until June 2022, with no language restrictions. The retrieval strategies included three core panels, associated with using AND connectors: (1) Hashimoto's thyroiditis, (2) Prevalence, and (3) Observational studies. The three core components are all composed of subject words and free words, which are retrieved from Pubmed's MeSH interface (Appendix 1).

The articles we included were all observational studies which reported the prevalence of HT with no interventions. We excluded articles for which full-text or original data were not available, and studies with a sample size fewer than 100 participants. Two authors (XH and YiS) independently screened eligible research records in accordance with title and abstract, respectively. And two additional authors (RT and YuS) retrieved the full text of potentially eligible articles to determine final inclusion. Inconsistent choices are resolved through discussion or third-party author participation.

### Data extraction and quality assessment

Data were extracted by one author using a standardized template, cross-checked by another author, and ambiguities resolved by discussion. Extracted data included the year of publication, first author, country of publication, geographic location, study type, sample size, diagnostic method, duration, sample source, and prevalence. We used an existing checklist modified by Hoy D to assess the quality of included studies (6). The list contained a total of nine questions, each question could be answered “yes” or “no,” and answering “yes” earns one point. The final score for each study was between zero and nine. Zero to three was low quality, four to six was medium quality, and seven to nine was high quality.

### Statistical analyses

We performed a meta-analysis of the extracted data and carried out statistical analysis using R software (version 4.0.3). The *meta* package in *R* software (version 4.0.3, Auckland University, USA) was mainly used for data analysis and the main outcome was assessed *via* single-arm analysis. For the prevalence or proportion, firstly, the normality test was conducted. If the data did not conform to the normality, it would be transformed by logarithm, logit, or double anti-sinusoidal transformation, and then, the inverse variance weighting method was used to combine.

The Cochrane *Q*-test and *I*^2^ value were used to test whether there was significant heterogeneity among all studies (7). According to the Meta-analysis of Observational Studies in Epidemiology guideline (8), if *P* > 0.10 and *I*^2^ ≤ 50%, it indicated that there was no statistical heterogeneity among the research results, and the fixed effect model was applied to analyze the results; If *P* ≤ 0.1 and *I*^2^ > 50%, the random effect model was used for meta-analysis. Publication bias was evaluated using *Egger's* test combined with a funnel plot. If there was obvious publication bias, we would use the trim-and-fill method to adjust for prospective plot asymmetry. And provided that necessary, sensitivity analysis was performed by grouping or omitting each study. Given that it was infeasible to make a quantitative synthesis and conduct a meta-analysis, a narrative approach and descriptive statistics were used. In addition, we performed subgroup analyses by income, geographic region, study type, diagnostic method, time of the study, and source of participants.

## Result

### Study characteristics

**Figure 1:**
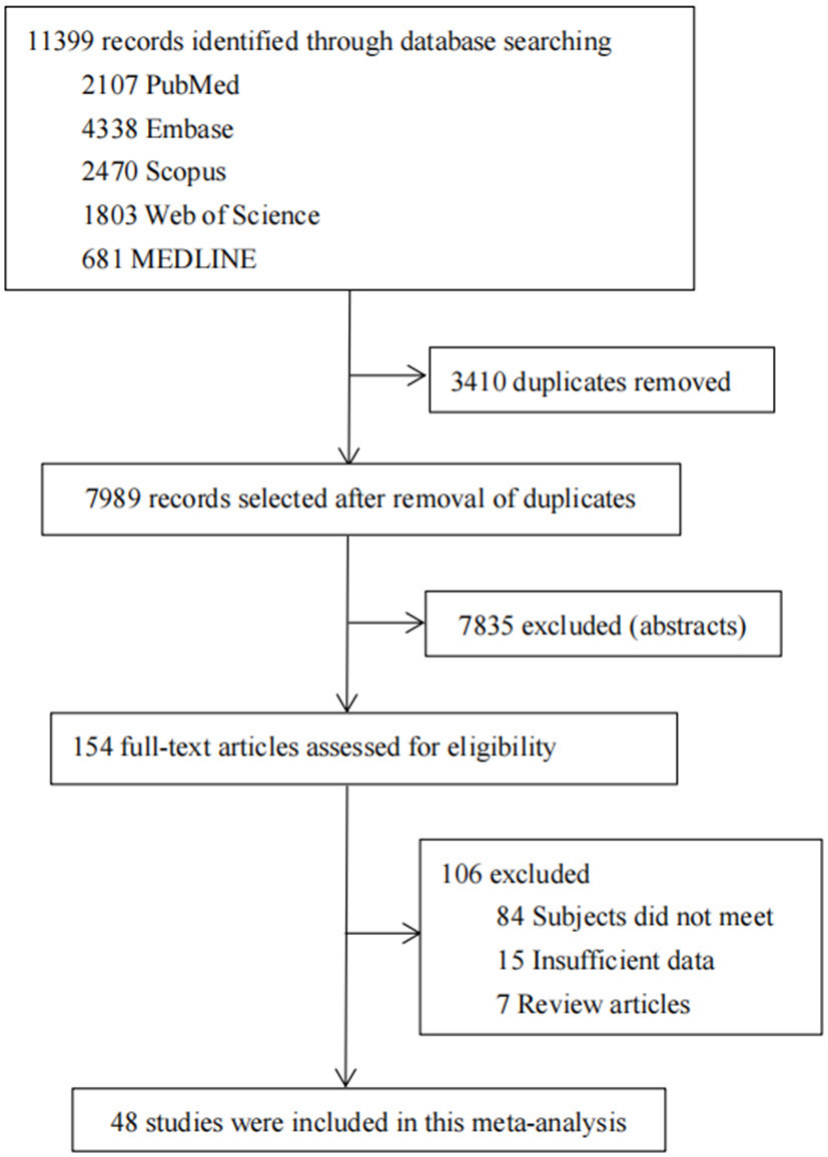
Study selection.

**Figure 2:**
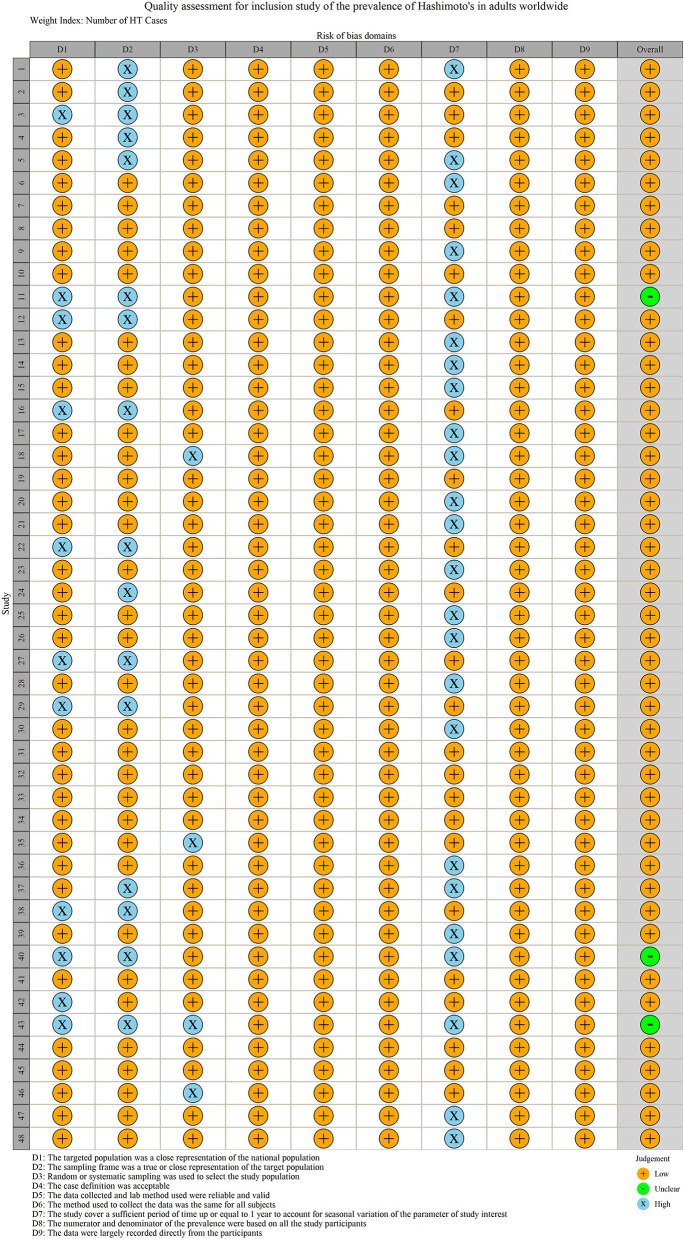
Literature quality assessment.

A total of 11,399 records were retrieved and identified across five databases. After removing duplicate literature, 7,989 articles were screened out. Hundred and fifty four studies were reviewed for full text, and a total of 48 studies were finally included (9–56) (Figure 1). All studies were observational (40 cross-sectional, eight cohort studies) involving 22,680,155 participants (Table 1). Thirty-seven of the 48 studies were population-based and 11 were clinical-based. Thirty-seven of the 48 studies were population-based and 11 were clinical-based. Twenty-seven studies confirmed HT was due to serum autoantibody levels, nine studies that used serum autoantibodies combined with ultrasonography to confirm the diagnosis, three studies that used serum autoantibodies to combine ultrasonography and fine needle aspiration, four studies on biopsy, only one study on ultrasonography alone, and four items not reported. Participants spanned from 1962 to 2021 and included studies published from 1966 to 2021 (Table 1). Twenty-one studies were published before 2000 and 27 studies were published after 2000. Of the 48 studies, 20 were from Europe, 16 from Asia, five from South America, three from North America, two from Africa, and two from Oceania, involving 19 countries. The quality scores of 48 articles are all six points or above, and the detailed quality assessment results are shown in Figure 2.

**Table 1:** Characteristics of included studies.

**Source**	**Year**	**Nation**	**Income group**	**Continent**	**Case**	**Total**	**Type of study design**	**Sample type**	**Test method**	**Time period**	**Sample source**
Okayasu I	1991	Japan	High income	Asia	328	1,826	Cross-sectional study	Thyroid tissue	Pathological section	1990	Clinic-based study
Okayasu I	1994	USA	High income	North America	457	2,040	Cross-sectional study	Thyroid tissue	Pathological section	1975-1992	Clinic-based study
Morinaka S	1995	Japan	High income	Asia	61	6,348	Cross-sectional study	Serum	Antibodies, ultrasound, fine needle aspiration	1990-1994	Clinic-based study
Tomimori E	1995	Brazil	Upper middle income	South America	72	547	Cross-sectional study	-	Ultrasound	1990-1995	Population-based study
Nagata K	1998	Japan	High income	Asia	142	1,039	Cross-sectional study	Serum	Antibody	1998	Population-based study
Aghini-Lombardi F	1999	Italy	High income	Europe	50	1,411	Cross-sectional study	Serum	Antibody	1998	Population-based study
Pedersen IB	2003	Denmark	High income	Europe	787	4,184	Array research	Serum	Antibody	1997-1998	Population-based study
Völzke H	2003	Germany	High income	Europe	47	3,941	Cross-sectional study	Serum	Antibody	1997-2001	Population-based study
Teng W	2006	China	Upper middle income	Asia	32	3,761	Cross-sectional study	Serum	Antibody	1999	Population-based study
Camargo RY	2006	Brazil	Upper middle income	South America	82	420	Cross-sectional study	Serum	Antibodies, ultrasound	1998-2005	Population-based study
Okosieme OE	2007	Nigeria	Lower middle income	Africa	7	104	Cross-sectional study	Serum	Antibody	2006	Clinic-based study
Kurata S	2007	Japan	High income	Asia	25	1,626	Cross-sectional study	Serum,thyroid tissue	Antibodies, ultrasound, fine needle aspiration	2002-2007	Clinic-based study
Teng X	2008	China	Upper middle income	Asia	67	778	Array research	Serum	Antibodies, ultrasound	2005	Population-based study
Camargo RY	2008	Brazil	Upper middle income	South America	183	1,085	Cross-sectional study	Serum	Antibody	2004	Population-based study
Döbert N	2008	Germany	High income	Europe	98	700	Cross-sectional study	Serum	Antibodies, ultrasound	2006	Population-based study
Benvenga S	2008	Italy	High income	Europe	4064	23,000	Array research	Serum,thyroid tissue	Antibodies, ultrasound, fine needle aspiration	1975-2005	Clinic-based study
Teng XC	2011	China	Upper middle income	Asia	363	3,813	Cross-sectional study	Serum	Antibodies, ultrasound	2007	Population-based study
Deshpande P	2016	Australia	High income	Oceania	17	198	Cross-sectional study	Serum	Antibody	1994	Population-based study
Fernando RF	2012	Sri Lanka	Lower middle income	Asia	353	5,200	Cross-sectional study	Serum	Antibody	2007-2008	Population-based study
Sardu C	2012	Italy	High income	Europe	678	25,885	Cross-sectional study	NR	NR	2009	Population-based study
Aghini Lombardi F	2013	Italy	High income	Europe	224	1,065	Cross-sectional study	Thyroid tissue	Antibodies, ultrasound	2010	Population-based study
Vecchiatti SM	2015	Brazil	Upper middle income	South America	106	4,613	Cross-sectional study	Thyroid tissue	Pathological section	2003-2007	Clinic-based study
Wu Q	2015	China	Upper middle income	Asia	172	6,152	Cross-sectional study	Serum	Antibody	2013	Population-based study
Flores-Rebollar A	2015	Mexico	Upper middle income	North America	36	427	Cross-sectional study	Serum	Antibodies, ultrasound	2010-2015	Population-based study
Li Y	2016	China	Upper middle income	Asia	187	2,856	Array research	Serum	Antibody	2013	Population-based study
Caturegli G	2016	USA	High income	North America	4	1,075	Cross-sectional study	NR	NR	2015	Population-based study
Tammaro A	2016	Italy	High income	Europe	2828	7,976	Array research	Serum	Antibody	2003-2010	Population-based study
Tolentino Júnior DS	2019	Brazil	Upper middle income	South America	85	60,413	Cross-sectional study	NR	NR	2016	Population-based study
Pilli T	2019	Italy	High income	Europe	9	142	Cross-sectional study	Serum	Antibody	2014-2019	Clinic-based study
Troshina EA	2021	Russia	Upper middle income	Europe	428	100,000	Cross-sectional study	Serum	Antibody	2018	Population-based study
Chen Y	2021	China	Upper middle income	Asia	298	2,946	Cross-sectional study	Serum	Antibodies,ultrasound	2016-2021	Population-based study
Kim HJ	2021	South Korea	High income	Asia	29429	217,05883	Array research	NR	NR	2002-2017	Population-based study
Yu ZW	2021	China	Upper middle income	Asia	148	1,159	Cross-sectional study	Thyroid tissue	Pathological section	2016-2020	Population-based study
Józków P	2017	Poland	High income	Europe	29375	586,703	Cross-sectional study	Serum	Antibody	2006-2013	Clinic-based study
Izic B	2021	Bosnia and Herzegovina	Upper middle income	Europe	358	82,000	Array research	Serum	Antibody	2015-2020	Population-based study
Gu F	2016	China	Upper middle income	Asia	17	5,293	Cross-sectional study	Serum	Antibodies, ultrasound	2011	Population-based study
Bjøro T	1984	Norway	High income	Europe	56	1,640	Cross-sectional study	Serum	Antibody	1979	Population-based study
Chabchoub G	2006	Tunisia	Lower middle income	Africa	246	1,079	Array research	Serum	Antibody	1990-2003	Clinic-based study
Dingle PR	1966	England	High income	Europe	52	469	Cross-sectional study	Serum	Antibody	1962	Population-based study
Jacobs A	1969	England	High income	Europe	99	989	Cross-sectional study	Serum	Antibody	1969	Population-based study
Tunbridge WM	1977	England	High income	Europe	56	2779	Cross-sectional study	Serum	Antibody	1972-1974	Population-based study
Prentice LM	1990	England	High income	Europe	124	698	Cross-sectional study	Serum	Antibody	1985-1990	Population-based study
Aho K	1971	Finland	High income	Europe	89	1,137	Cross-sectional study	Serum	Antibody	1970	Clinic-based study
Gordin A	1972	Finland	High income	Europe	282	2,961	Cross-sectional study	Serum	Antibody	1967-1972	Population-based study
Bryhni B	1996	Norway	High income	Europe	176	2,551	Cross-sectional study	Serum	Antibody	1979-1980	Population-based study
Konno N	1993	Japan	High income	Asia	457	4,110	Cross-sectional study	Serum	Antibody	1990-1991	Population-based study
O'Leary PC	2005	Australia	High income	Oceania	262	2,115	Cross-sectional study	Serum	Antibody	1981	Population-based study
Li YS	2008	China	Upper middle income	Asia	353	3,018	Cross-sectional study	Serum	Antibodies, ultrasound	2004	Population-based study

### Meta-analysis results

**Figure 3:**
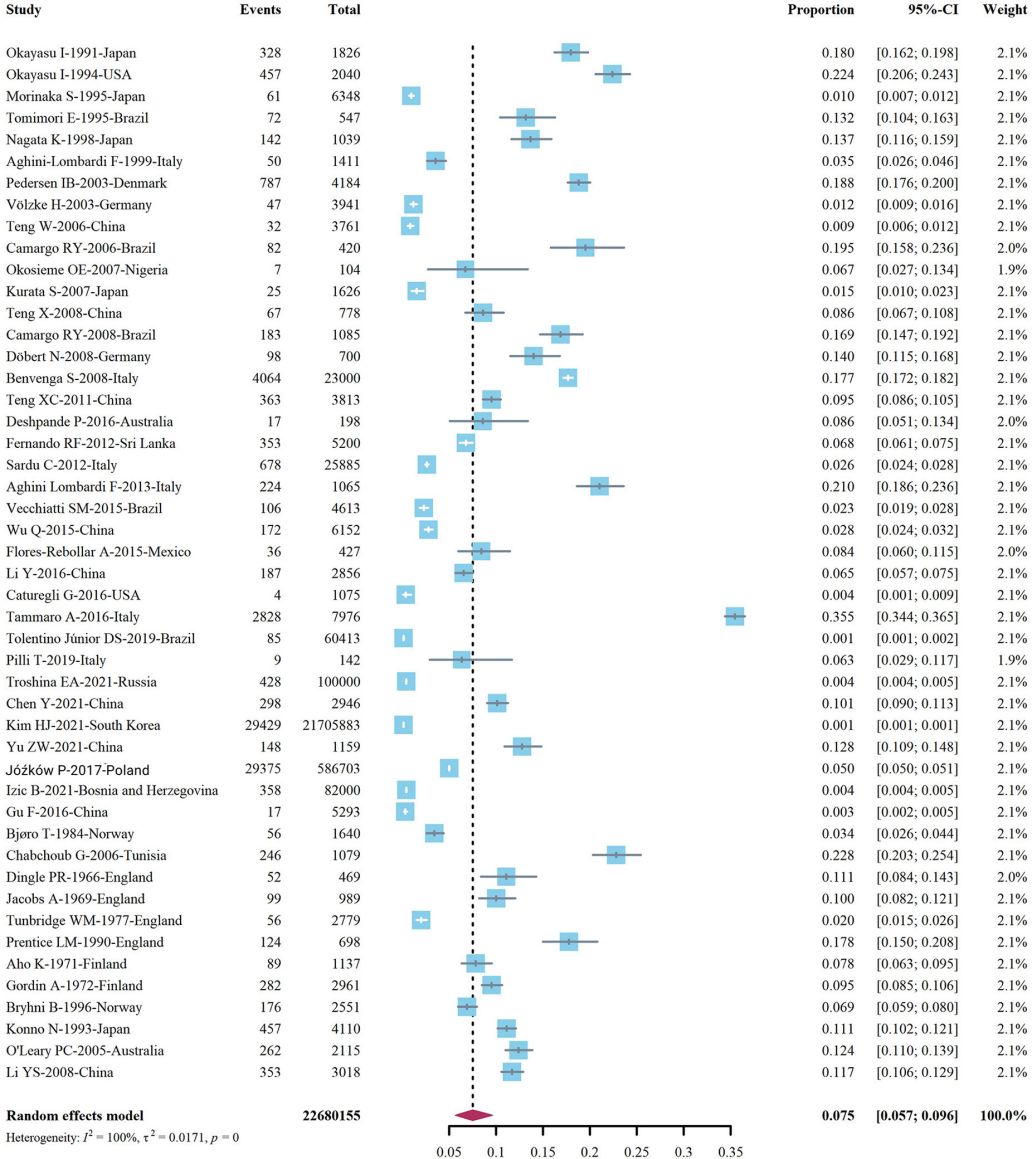
Global prevalence of Hashimoto's thyroiditis.

**Figure 4:**
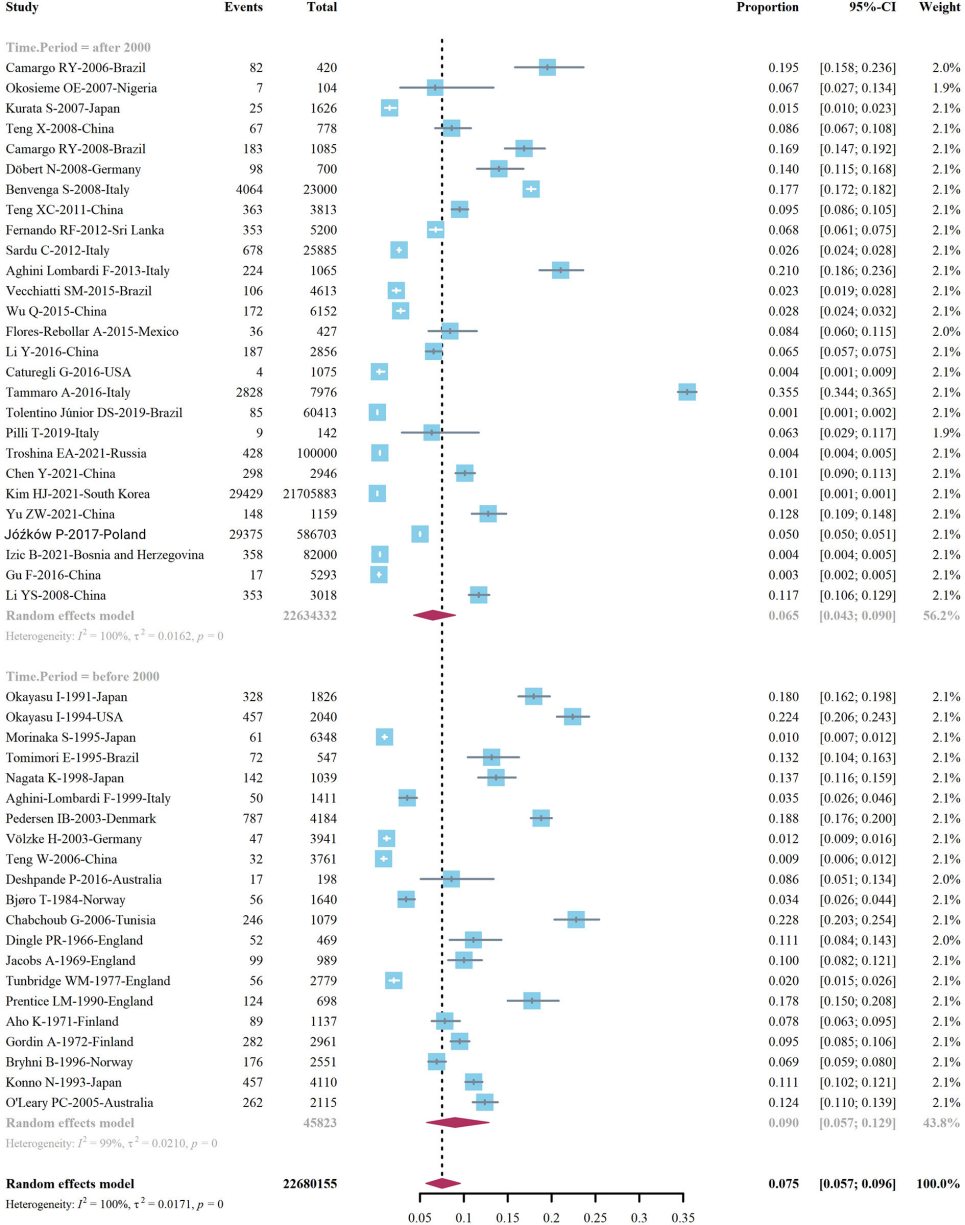
Global prevalence of Hashimoto's thyroiditis, by time of study implementation.

**Figure 5:**
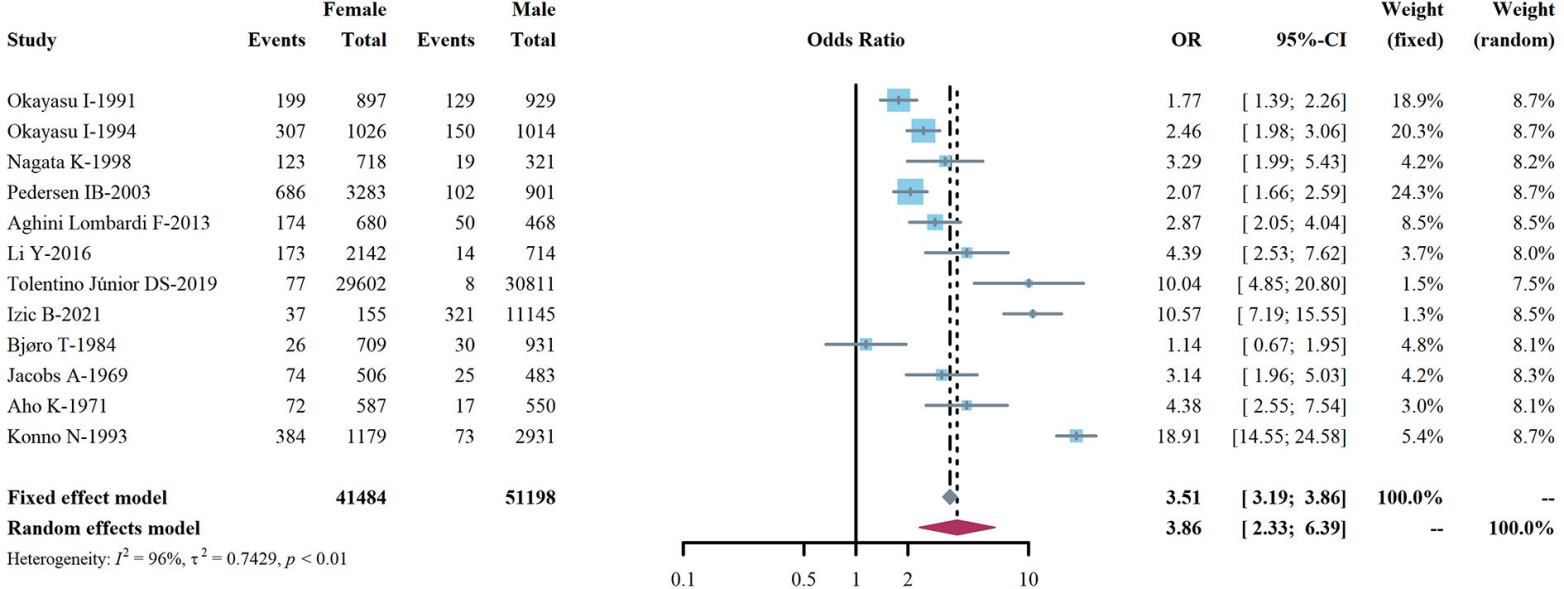
Global prevalence of Hashimoto's thyroiditis, by sex.

**Figure 6:**
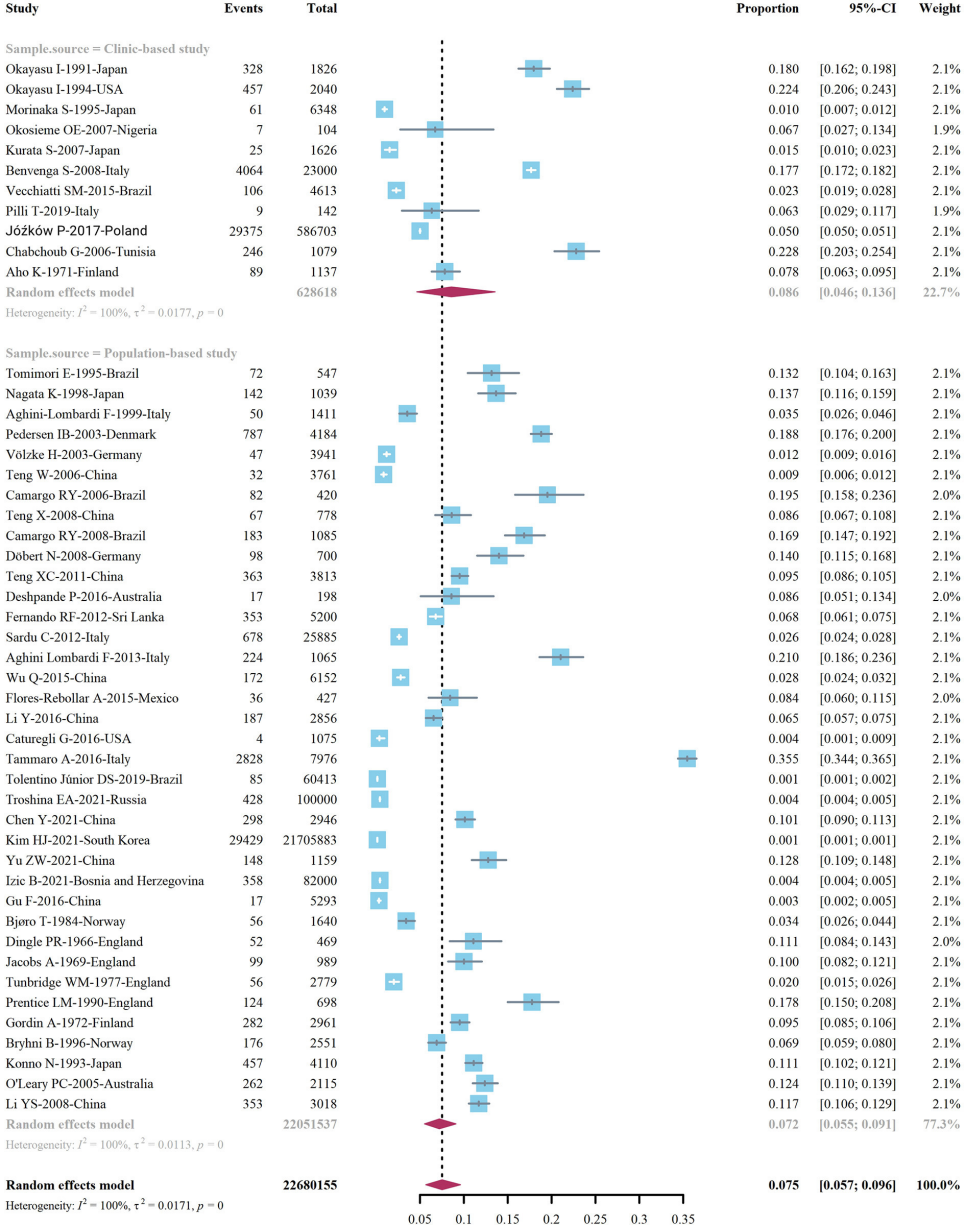
Global prevalence of Hashimoto's thyroiditis, by study source.

**Figure 7:**
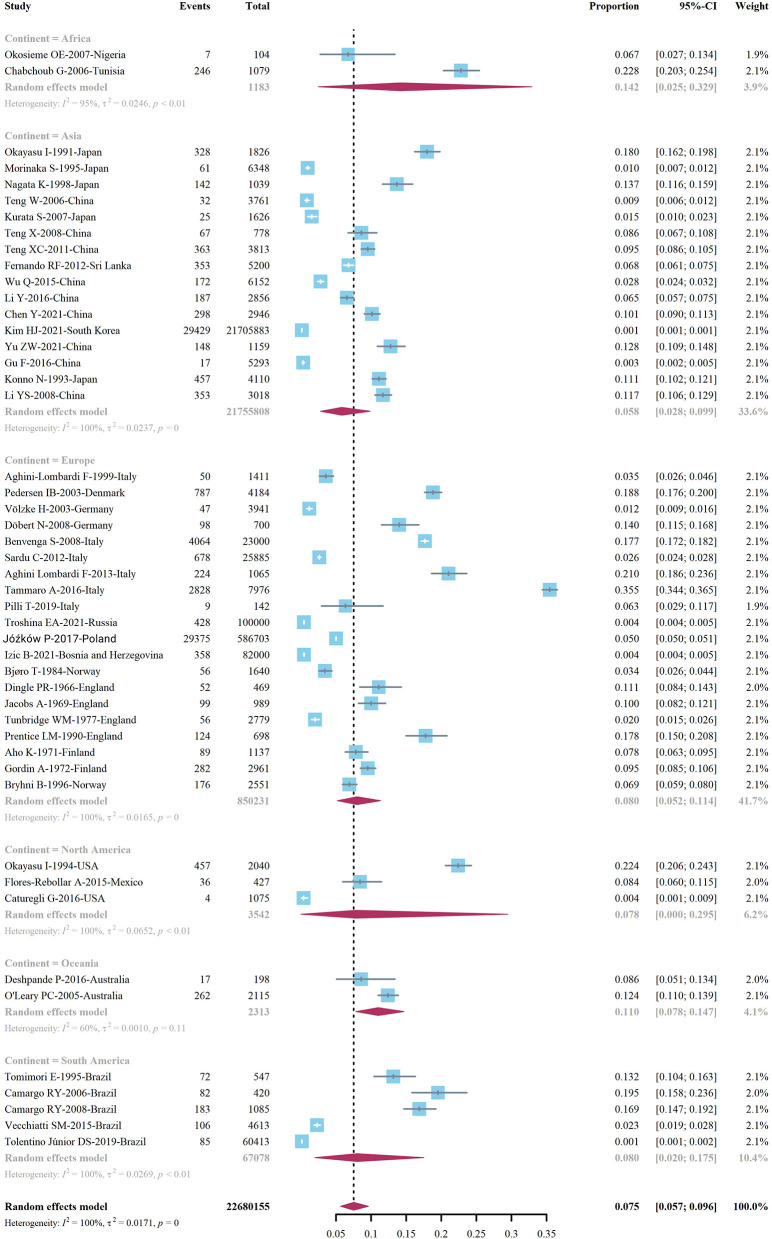
Global prevalence of Hashimoto's thyroiditis, by geographic location.

**Figure 8:**
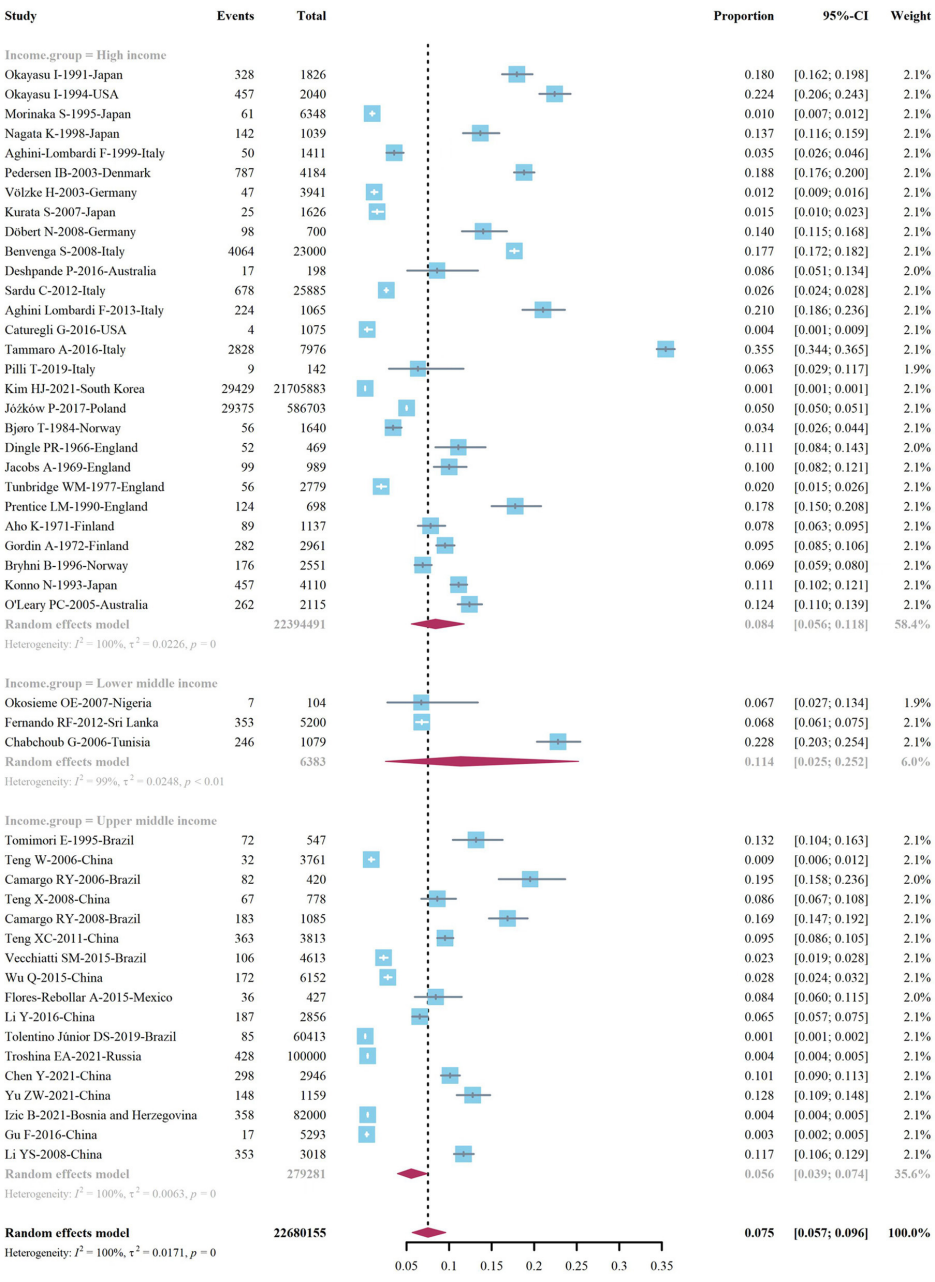
Global prevalence of Hashimoto's thyroiditis, by income.

We extracted data points from 48 studies that met the inclusion criteria to form the initial data. The initial data was tested by Shapiro-Wilk (SW), *P* = 0.002047 < 0.05, which did not conform to the normal distribution, so we adopted Freeman-Tukey double arcsine transformation. After the transformation, the data showed a normal distribution. We executed a data pooled meta-analysis and estimated the global prevalence of HT in adults to be 7.5% (Figure 3). The prevalence of HT in adults has declined over the past 60 years, from 9% before 2000 to 6.5% after 2000 (Figure 4). HT prevalence of female adults was 3.86 adult males times (17.5 vs. 6.0%) (Figure 5). The prevalence of HT in clinical studies was 8.6% higher than in population-based studies (8.6 vs. 7.5%) (Figure 6). The prevalence of HT in adults from different continents was diverse, with the highest prevalence of HT in African adults (14.2%), followed by Oceania (11.0%), 8.0% in both South American and European adults, and 7.8% in North America. The lowest prevalence of HT in Asian adults was 5.8% (Figure 7). According to the latest income classification of the World Bank (https://datatopics.worldbank.org/world-developmentindicators/the-world-by-incomeand-region.htm), we conducted a subgroup analysis according to low-income, lower-middle-income, upper-middle-income, and high-income groups. The results showed that the prevalence of HT adults in the low-middle-income group was 11.4%, in the upper-middle-income group, the prevalence was 5.6%, and in the high-income group, the prevalence was 8.4% (Figure 8).

### Quality assessment

**Figure 9:**
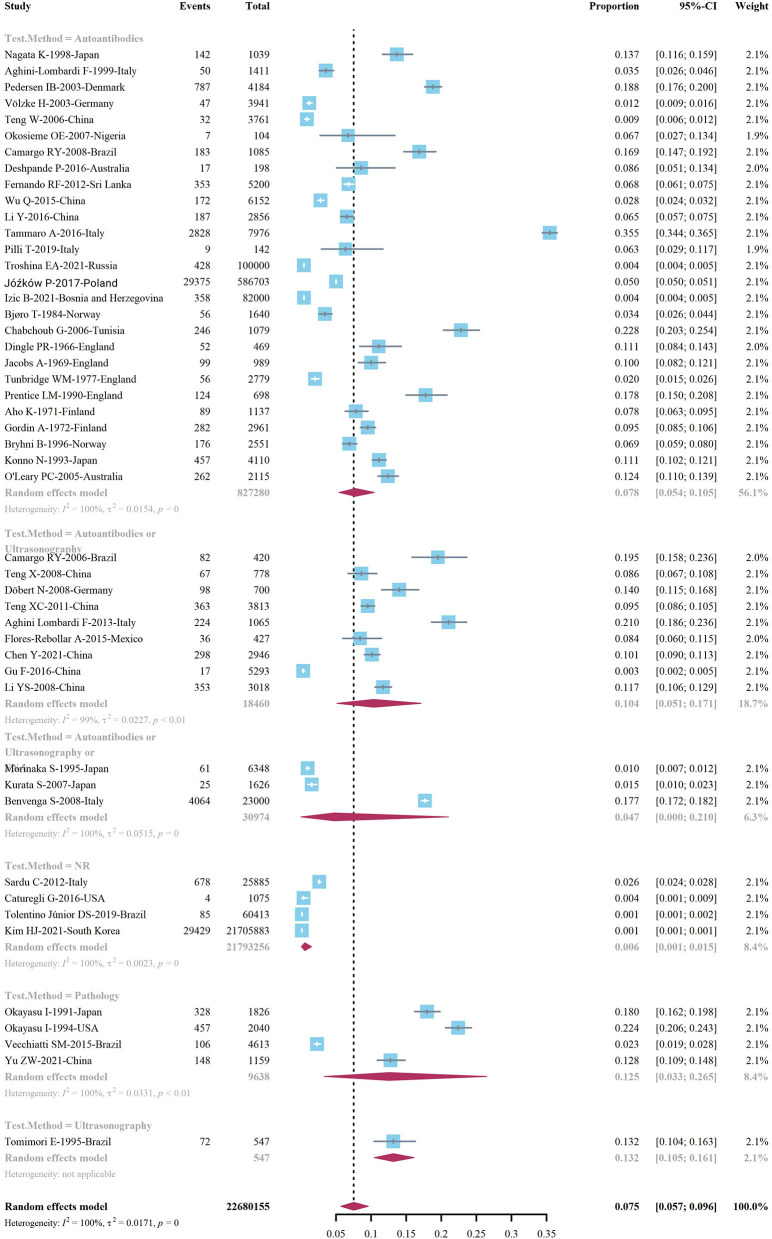
Subgroup analysis of diagnostic methods.

We conducted a subgroup analysis according to different diagnostic methods. The results of analysis indicated that the prevalence of HT diagnosed by serum autoantibodies was 7.8 (95% Cl 5.4–10.5%), and the prevalence of HT diagnosed by ultrasonography was 13.2 (95% Cl 5.7–9.6%). Similarly, the prevalence of HT was diagnosed by pathological examination was 12.5 (95% Cl 3.3–26.5%) (Figure 9). The prevalence rate of HT diagnosed by serum autoantibody level combined with color Doppler ultrasound was 10.4 (95% Cl 5.1–17.1%), while the prevalence rate of HT diagnosed by serum autoantibody level, color Doppler ultrasonography, and fine needle aspiration (FNA) was 4.7 (95% 0.0–21.0%) (Figure 9). We performed a subgroup analysis of samples diagnosed with HT. The prevalence of HT confirmed by serum was 7.8 (95% Cl 5.8–10.1%), the prevalence of HT confirmed by serum or pathological tissue was 7.6 (95% Cl 0.0–30.2%), and the prevalence of HT confirmed by thyroid tissue alone was 14.1 (95% Cl 5.2–26.5%).

**Figure 10:**
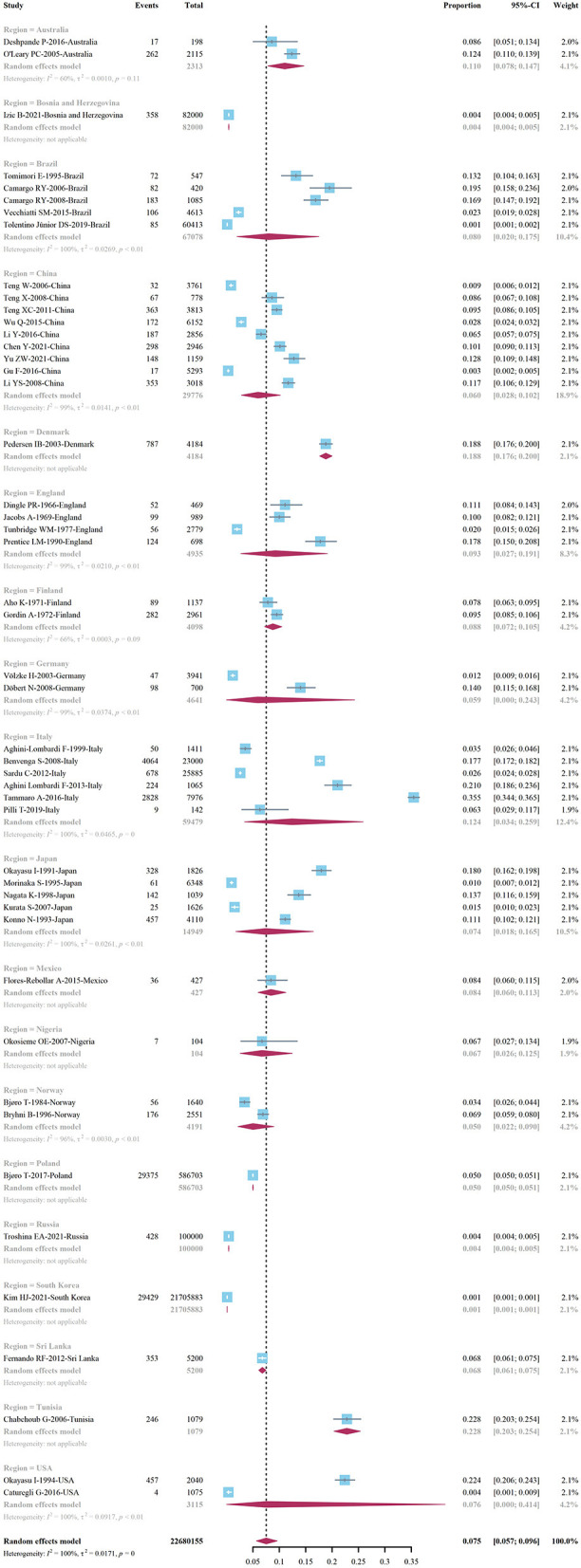
Prevalence of Hashimoto's thyroiditis by country.

The prevalence of HT in adults varies significantly across countries, ranging as high as 22.8 (95% Cl 20.3–25.4%) in Tunisia and 18.8 (95% Cl 17.6-−20.0%) in Denmark, while the prevalence in South Korea is as low as 0.1%, and in adults in Russia and Bosnia and Herzegovina, The prevalence of HT was 0.4% (Figure 10).

**Figure 11:**
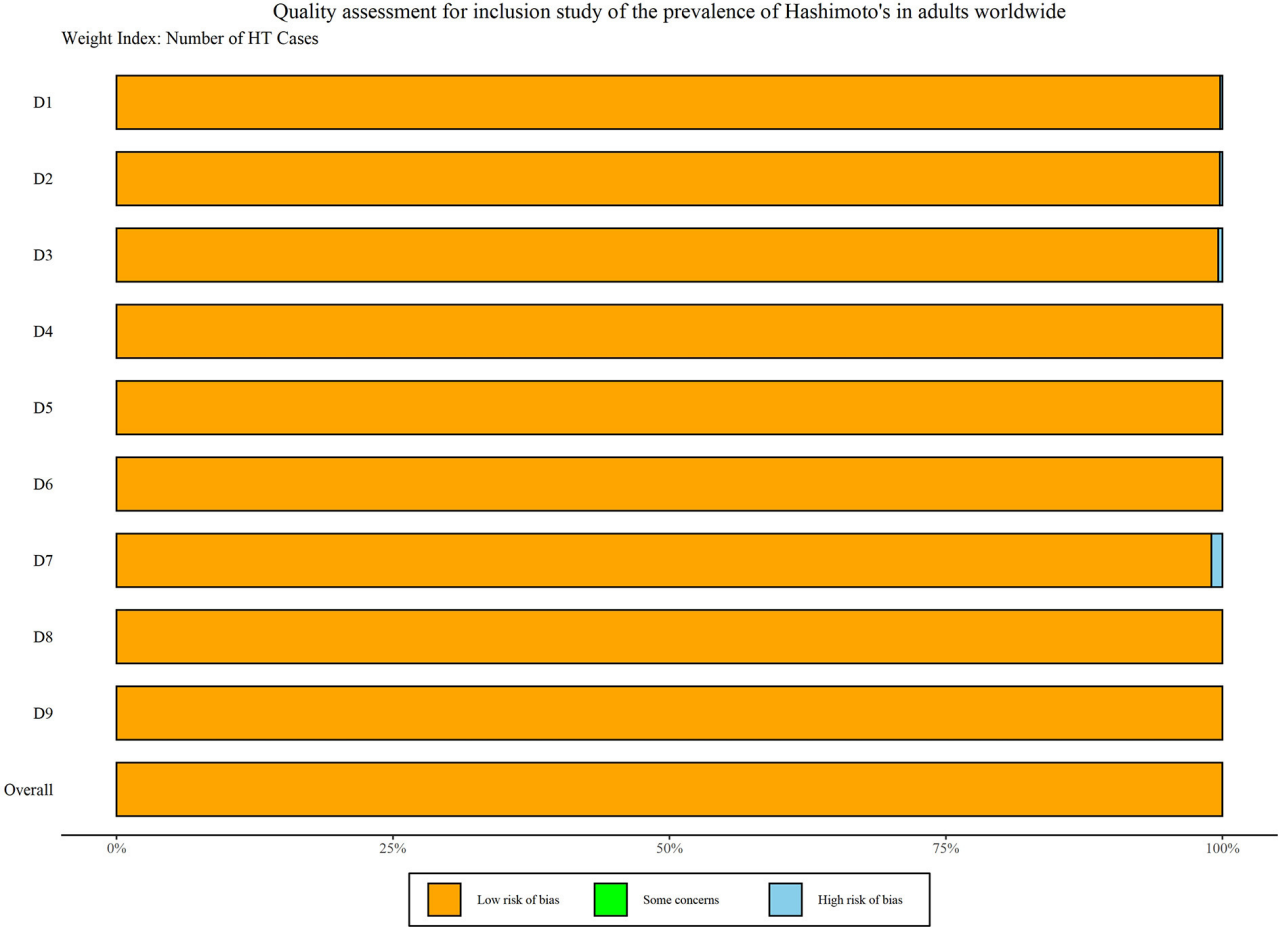
Literature quality risk assessment chart.

**Figure 12:**
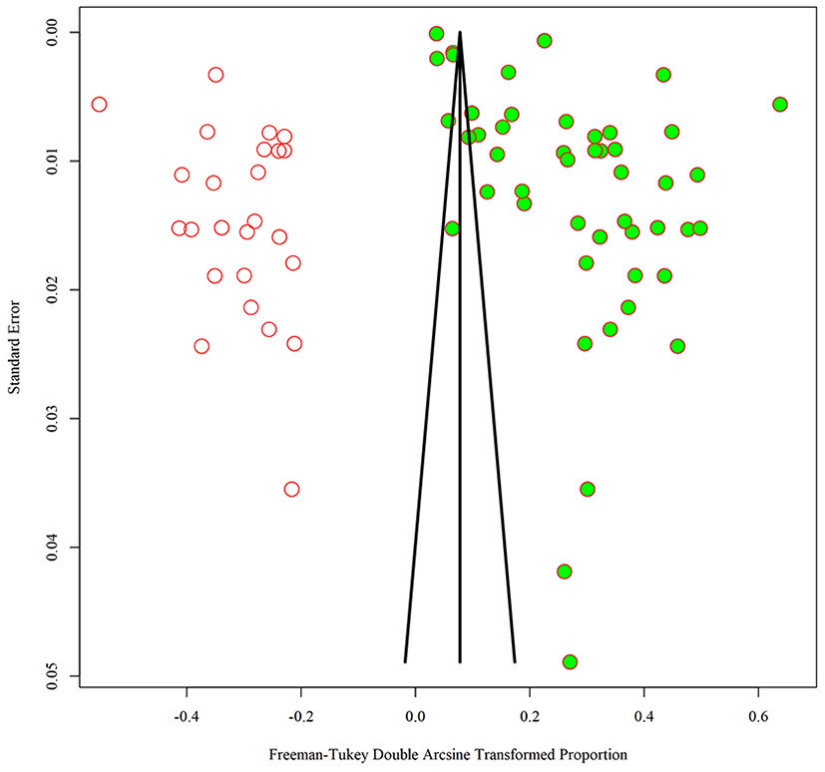
Trim-and-fill method to adjust for funnel plot asymmetry.

**Figure 13:**
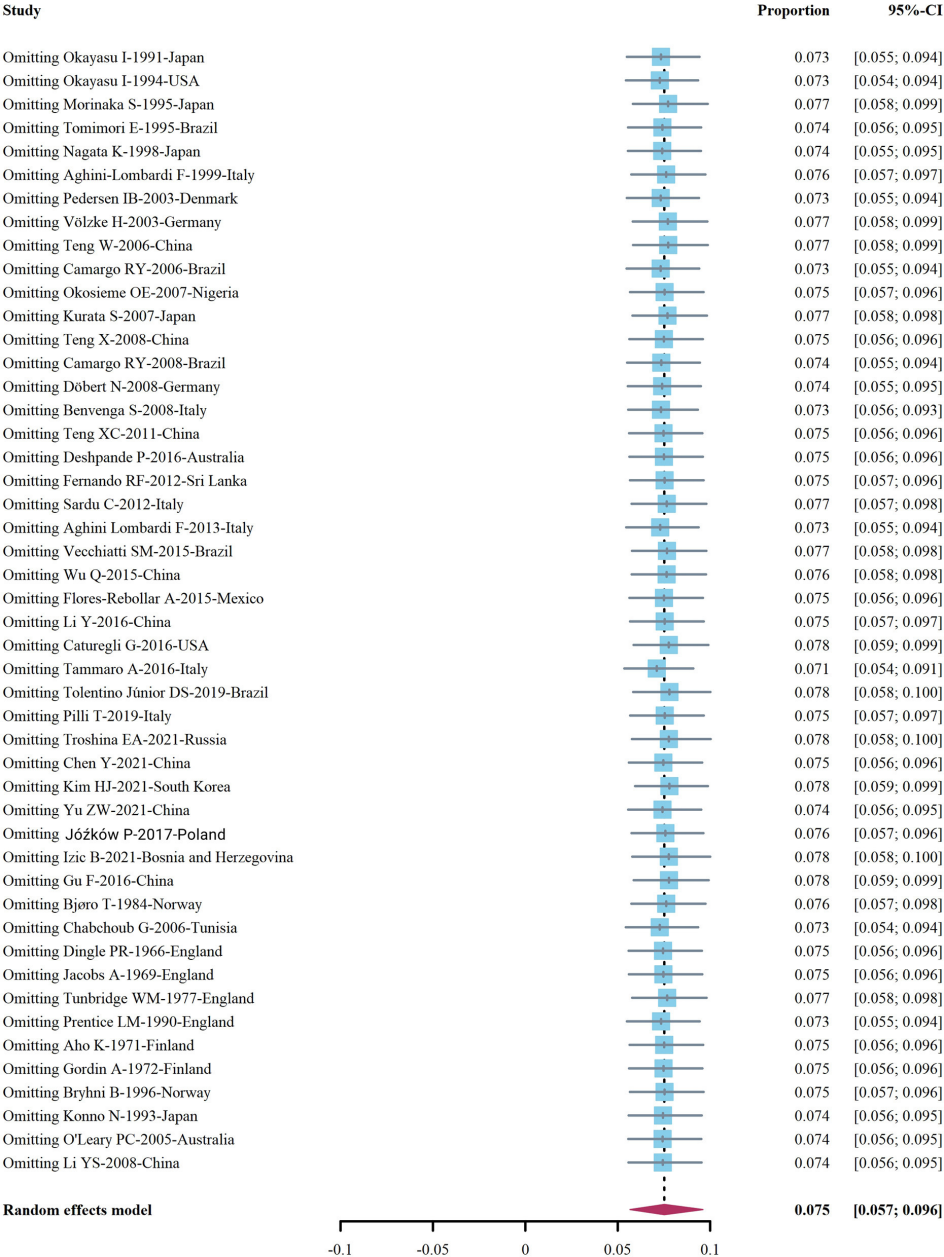
Sensitivity analysis chart of included studies.

Among the 48 observational studies, 42 studies were low risk (good quality), and 6 studies were rated as medium risk, making a risk of bears evaluation chart (Figure 11). Funnel plots and Egger's test linear regression were used to test for publication bias. There was a publication bias in each study (*P* < 0.05). The results are shown in the Appendix 1 (Figure 12). We used the trim-and-fill method to correct for publication bias. Sensitivity analysis showed that the literature included in this study had little impact on the results of the study analysis, and removing any one of the studies would have a small impact (Figure 13).

## Discussion

This systematic review and meta-analysis provides a comprehensive assessment of the prevalence of HT in adults worldwide, which is associated with the occurrence of various malignant tumors (2, 57, 58). In total, this study pooled 48 studies involving more than 20 million adult patients with HT and performed subgroup analyses by type of study, diagnostic method, the timing of study conduct, patient source, gender, geographic location, and economic level. We found that the prevalence of HT in adults varies widely across continents. African adults have the highest prevalence of HT, more than double the prevalence in Asia. We also found that the prevalence of HT in adults decreased as time went by. Unlike previous studies (59–61), the prevalence of Hashimoto's thyroiditis in adults decreased over time.

In our study, the overall prevalence of HT in adults was 7.5%, with a prevalence of 17.5% in women and 6.0% in men. The risk of developing HT in adult women is approximately 4 times than that of adult men. Tunbridge et al. reported that in the United States, 10% of the population had thyroid antibodies, a prevalence of 14% in whites and about 5% in blacks (49). In the report, the prevalence of female HT was higher, and the ratio of female HT patients to male HT patients was 8–9:1 (60). In contrast, the prevalence of HT in the study by Gu et al. was much lower (3.2%) (24).

Our study involved 19 countries and 6 continents (Europe, Asia, South America, North America, Africa, and Oceania), and the prevalence data of HT adults in various regions of the world were aggregated to estimate the prevalence of HT in adults and development trend as far as possible. From 1960 to the present, we pooled data on adults with HT every 20 years to assess trends in the prevalence of HT in adults. We were surprised to find that the prevalence of HT in adults decreased regardless of whether we divided the study by time before and after 2000 or every 20 years. This is inconsistent with most studies. This may be related to different regions and periods, socioeconomic development, availability, and availability of medical resources. The progress of the social economy will promote people to pay more attention to physical health, increase the records of hospital visits, and also increase the chances of HT being detected and recorded. Therefore, studies in different periods in the same region have shown an increase in the prevalence of HT. Whether this is due to an increase in the prevalence of HT due to an increase in the number of people with HT or an increase in the probability of being detected is unclear. In our study, which pooled population-based and clinical-based studies separately, the prevalence of HT in adults was lower in population-based studies than in clinical studies (7.2 vs. 8.6%). This provides persuasive evidence for our hypothesis. Another reason may be that in the studies we included, the proportion of studies in different regions and different periods was different. The lowest prevalence of HT in adults (6.7%) was observed in 2000–2021, with a higher proportion of studies in Asia and Europe during this period, and a relatively high proportion of HT studies in North and South America between 1960–1981.

When we performed a subgroup analysis according to the latest income classification of the World Bank, we found an interesting phenomenon. A higher prevalence of HT among adults in low- and middle-income countries is conceivable. However, the higher prevalence of HT in adults in high-income countries than in upper-middle-income countries is indeed an interesting finding. This may be related to the pathogenesis of HT. People living in economically developed countries have increased pressure from various aspects (62), and mental health status is also an important cause of HT (61).

In our systematic review, Africa had the highest prevalence (14.2%) while Asia had the lowest prevalence (5.8%). The prevalence varies widely, which may be related to lifestyle and dietary habits. The pathogenesis of Hashimoto's thyroiditis is still unclear, and some studies have pointed out that the lack of micronutrients may be related to the pathogenesis of thyroiditis, such as vitamin D deficiency (4, 63). In Africa, some people still live a traditional way of life (gathering, hunting, nomadic animals) (64). The lack of diversification of nutrient intake due to geographic location, environmental factors, and economic level may explain the high prevalence of HT in African adults. It may also be related to the number of studies included, with 16 studies included in Asia and only two studies from Africa. We cannot rule out differences due to differences in the number of included cases. HT is a chronic inflammatory disease, and chronic inflammation increases the risk of a variety of malignancies (65–67). The studies we included were all observational studies, most of which were cross-sectional studies, only to assess the prevalence of HT, with no follow-up for later cancer risk and treatment in HT patients. Therefore, our pooled estimates of the adult prevalence of HT may underestimate the actual burden on health care.

Our study systematically evaluated the global adult prevalence of HT for the first time and includes the largest number of studies on the prevalence of HT in adults. However, our study also involves certain limitations. The period of the studies we included was large. From 1962 to 2021, there may exist differences in the diagnostic criteria and detection methods of HT in different periods. In many cases, we were unable to obtain specific information on the diagnostic criteria for HT in the studies. Although we were unable to unify the diagnostic criteria for HT, we analyzed the prevalence of HT in adults by detection method. There was considerable heterogeneity among studies, and we performed subgroup analyses where possible, but this did not reduce heterogeneity between studies. This may be related to the regional economy, dietary habits, lifestyle, and diagnostic criteria of HT in different periods of the included studies. Sensitivity analysis showed that each study included in the study had little effect on the results and had good stability. The studies we included may have some selection bias, but the information in the studies was insufficient to assess these errors. We hope that the diagnostic criteria for HT will be unified as much as possible in future research so that the research results will be more convincing.

## Conclusions

In conclusion, we found that the prevalence of Hashimoto's thyroiditis in adult females is approximately four times that of male patients, and the prevalence of HT is relatively high in adults worldwide, especially in Africa. There are differences in the prevalence of HT among adults at different economic levels. The prevalence of HT in low- and middle-income countries is the highest, and the prevalence in high-income countries is higher than that in upper-middle-income countries. Therefore, we suggest that public health departments in low- and middle-income countries should take strategic measures to prevent, detect, and treat HT as early as possible, while high-income countries should also pay attention to the prevalence of HT and the burden of medical services.

## Data availability statement

The original contributions presented in the study are included in the article/supplementary material, further inquiries can be directed to the corresponding author/s.

## Author contributions

XH and YShen conceptualized, involved, and conducted this study. XH wrote the first draft under the guidance of HQ. YC, YShen, YSheng, and RT reviewed drafts and provided input for all versions. XH and YC accessed, verified, analyzed, and interpreted the data. All authors contributed to the article and approved the submitted version.

## Funding

This project was supported by the Shanghai Key Clinical Specialty Construction Project of China (shslczdzk03801).

## Conflict of interest

The authors declare that the research was conducted in the absence of any commercial or financial relationships that could be construed as a potential conflict of interest.

## Publisher's note

All claims expressed in this article are solely those of the authors and do not necessarily represent those of their affiliated organizations, or those of the publisher, the editors and the reviewers. Any product that may be evaluated in this article, or claim that may be made by its manufacturer, is not guaranteed or endorsed by the publisher.

## Appendix

Literature search strategies.

**Pubmed:** ((((epidemiology[Title/Abstract])) OR (incidence[Title/Abstract])) OR (prevalence[Title/Abstract])) AND ((”Hashimoto Disease“[Mesh]) OR ((((((((((((((((((((((((Disease, Hashimoto[Title/Abstract])) OR (Hashimoto Struma[Title/Abstract])) OR (Hashimoto Thyroiditis[Title/Abstract])) OR (Hashimoto Thyroiditides[Title/Abstract])) OR (Thyroiditides, Hashimoto[Title/Abstract])) OR (Thyroiditis, Hashimoto[Title/Abstract])) OR (Hashimoto's Syndrome[Title/Abstract])) OR (Hashimoto Syndrome[Title/Abstract])) OR (Hashimoto's Syndromes[Title/Abstract])) OR (Hashimotos Syndrome[Title/Abstract])) OR (Syndrome, Hashimoto's[Title/Abstract])) OR (Syndromes, Hashimoto's[Title/Abstract])) OR (Hashimoto's Struma[Title/Abstract])) OR (Chronic Lymphocytic Thyroiditis[Title/Abstract])) OR (Chronic Lymphocytic Thyroiditides[Title/Abstract])) OR (Lymphocytic Thyroiditides, Chronic[Title/Abstract])) OR (Lymphocytic Thyroiditis, Chronic[Title/Abstract])) OR (Thyroiditides, Chronic Lymphocytic[Title/Abstract])) OR (Thyroiditis, Chronic Lymphocytic[Title/Abstract])) OR (Hashimoto's Disease[Title/Abstract])) OR (Disease, Hashimoto's[Title/Abstract])) OR (Hashimotos Disease[Title/Abstract])) OR (Autoimmune thyroiditis[Title/Abstract]))).
